# Pharmacokinetic and Metabolism Studies of Curculigoside C by UPLC-MS/MS and UPLC-QTOF-MS

**DOI:** 10.3390/molecules24010021

**Published:** 2018-12-21

**Authors:** Di Wu, Han Wang, Jing Tan, Cuizhu Wang, Hongqiang Lin, Hailin Zhu, Jinping Liu, Pingya Li, Jianyuan Yin

**Affiliations:** School of Pharmaceutical Sciences, Jilin University, Fujin Road 1266, Changchun 130021, China; dwu15@mails.jlu.edu.cn (D.W.); hanw17@mails.jlu.edu.cn (H.W.); tanjing17@mails.jlu.edu.cn (J.T.); wangcz15@mails.jlu.edu.cn (C.W.); linhq17@mails.jlu.edu.cn (H.L.); 13578965875@163.com (H.Z.); liujp@jlu.edu.cn (J.L.); lipy@jlu.edu.cn (P.L.)

**Keywords:** curculigoside C, UPLC-MS, pharmacokinetics, metabolism

## Abstract

Pharmacokinetic and metabolism studies were carried out on curculigoside C (CC), a natural product with good antioxidant and neuroprotective effects, with the purpose of investigating the effects of the hydroxyl group at C-3′ in curculigoside. A rapid and sensitive method with UPLC-MS was developed and fully validated for the first time in the pharmacokinetic analysis for quantification of CC in rat plasma. The assay was linear (R^2^ > 0.9984) over the concentration range of 1–2500 ng/mL, with the lower limit of quantification (LLOQ) being 1 ng/mL. The intra-day and inter-day precision (expressed as relative standard deviation, RSD) ranged from 4.10% to 5.51% and 5.24% to 6.81%, respectively. The accuracy (relative error, RE) ranged from −3.28% to 0.56% and −5.83% to −1.44%, respectively. The recoveries ranged from 92.14% to 95.22%. This method was then applied to a pharmacokinetic study of rats after intragastric administration of 15, 30 and 60 mg/kg CC. The results revealed that CC exhibited rapid oral absorption (*T_max_* = 0.106 h, 0.111 h, and 0.111 h, respectively), high elimination (*t*_1/2_ = 2.022 h, 2.061 h, and 2.048 h, respectively) and low absolute bioavailability (2.01, 2.13, and 2.39%, respectively). Furthermore, an investigation on the metabolism of CC was performed by UPLC-QTOF-MS^E^. Twelve metabolites of CC from plasma, bile, urine and faeces of rats were confirmed. The main metabolic pathways of CC, which involve dehydration, glucosylation, desaturation, formylation, cysteine conjugation, demethylation and sulfonation, were profiled. In conclusion, this research has developed a sensitive quantitative method and demonstrated the metabolism of CC in vivo.

## 1. Introduction

Curculiginis rhizoma, the dried rhizome of *Curculigo orchioides* Gaertn, is rich in phenolic glycosides [1,2]. Among the phenolic glycosides, curculigosides have been demonstrated to be the major components responsible for the pharmacological effects of Curculiginis rhizoma [3,4,5,6]. Several curculigosides have been isolated from Curculiginis rhizoma so far, such as curculigosides A, -B, -C, -D, -E, -F, -G and -H [7,8,9]. Due to its highest content in Curculiginis rhizoma, curculigoside A has been the main curculigoside studied for its pharmacological effects [10,11,12,13] and pharmacokinetic profiles [14,15]. 

Curculigoside C (CC) is the natural product of C-3′ hydroxylation of curculigoside A [16]. It was reported that CC has better antioxidant and neuroprotective effects that the parent compound [17,18]. For example, in the assay on hydroxyl radicals produced by H_2_O_2_/Fe^2+^, CC exhibited more significant scavenging effects than curculigoside A, and the scavenging effect of CC was comparable with that of pigallocatechin gallate, a known antioxidant [17]. Moreover, in the 1,1-diphenyl-2-picrylhydrazyl (DPPH) radical scavenging assay, CC also showed better antioxidant activity than curculigoside A, and had a comparable IC_50_ value to vitamin C, another well-known antioxidant [18]. Furthermore, in the evaluation of salvaging SY5Y cell death induced by H_2_O_2_, CC displayed better neuroprotective effect than curculigoside A [18]. The discussion about the structure- activity relationship showed that the presence of OH at C-3′ and the two vicinal oxygen-bearing groups at the benzene ring might be the functional groups [18]. 

It is known that the pharmacokinetic parameters are necessary for the evaluation of active ingredients [19,20]. To the best of our knowledge, there are no reports on pharmacokinetic assays of CC. In the present research, CC was used for pharmacokinetic and metabolism studies. Due to the high sensitivity and rapid quantification [21,22], an ultra-high performance liquid chromatography combined with tandem quadrupole mass spectrometry (UPLC-MS/MS) method was developed and validated for the determination of CC in rat plasma. This method was then applied to pharmacokinetic study of CC in rats. In addition, the metabolic characteristics of CC were also studied to elucidate the dynamic process of CC in rats. The aims were to investigate the pharmacokinetics and metabolic characteristics of CC with a hydroxyl group at C-3′. In summary, this exploration provides a sensitive quantitative assay method and illustrated the pharmacokinetic characteristics of CC in rats.

## 2. Results

### 2.1. Pharmacokinetic Study

#### 2.1.1. Method Development

**Figure 1:**
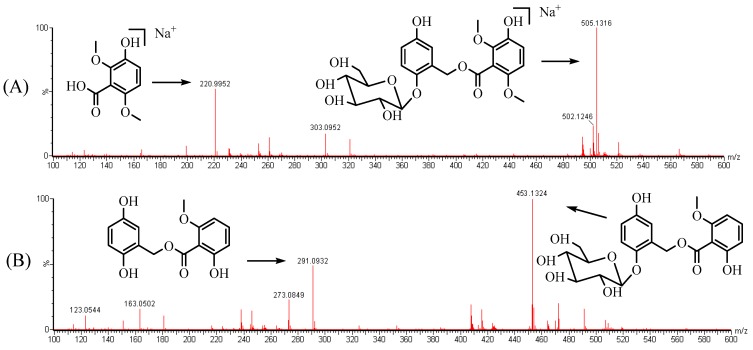
Mass spectra and the proposed fragmentation patterns of CC (**A**) and the internal standard (IS) (**B**).

Aiming at increasing the extraction recovery and minimizing the matrix effect, different sample pre-treatment methods such as solid-phase extraction, liquid-liquid extraction, or protein precipitation were comparatively investigated. As a consequence, the optimized one-step protein precipitation using methanol was selected. Several additions were tested to improve the sensitivity of the CC and curculigoside B (IS), 0.1% formic acid was chosen. No significant signal diminution or enhancement was found under the current conditions used in this research. The IS has a similar structure, chromatographic behavior and extraction efficiency to those of CC and both of them showed strong mass responses in positive ESI mode. ESI^+^ showed that CC (MW: 482.1) and IS (MW: 452.1) formed predominately molecular ions [M + Na]^+^ at *m*/*z* 505.1 and mainly deprotonated molecular ions [M + H]^+^ at *m*/*z* 453.1 in full-scan product ion spectra, respectively. Precursor ion and product ions were selected according to the stability and ion response. Mass spectrometric scanning was operated on multiple reaction monitoring (MRM) using follow monitored transitions: *m*/*z* 505.1 → 221.0 for CC, *m*/*z* 453.1 → 291.1 for IS, respectively. The f ragmentation pathways of CC and the IS are shown in Figure 1. 

#### 2.1.2. Method Validation

**Figure 2:**
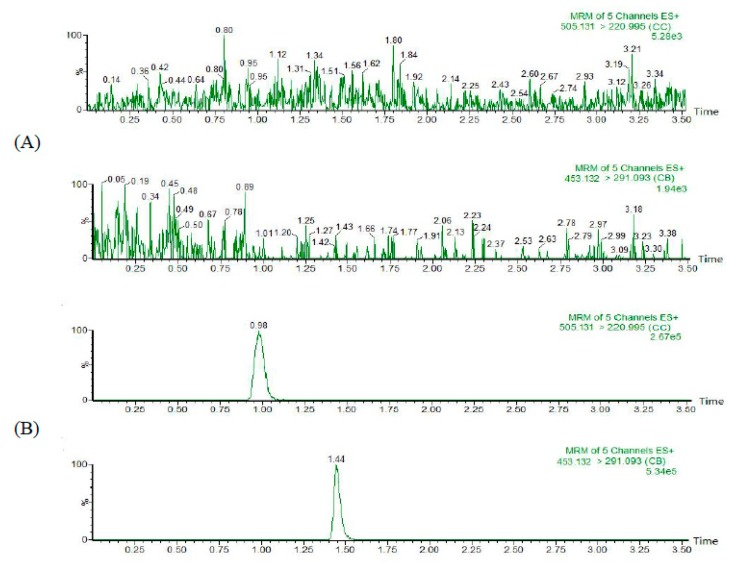

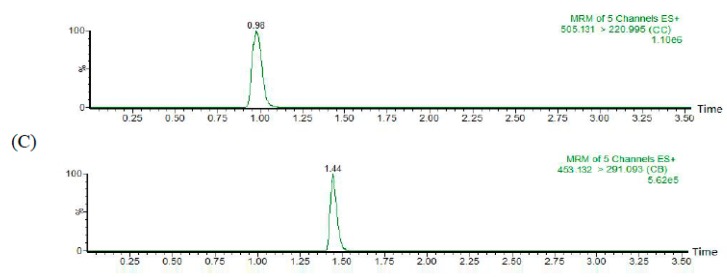
Typical chromatograms of blank plasma (**A**), blank plasm (**B**) spiked with CC and IS, and plasma sample (**C**) collected at an oral dose of 60 mg/kg CC.

The detection of CC (RT, 0.98 min) and IS (RT, 1.44 min) showed high selectivity. No significant interference of endogenous plasma components was observed at the retention time of CC and the IS. Typical chromatograms of blank plasma (A), blank plasma spiked with CC at 100 ng/mL and IS at 100 ng/mL (B), and plasma sample collected from a rat at 0.083 h after an oral dose of 60 mg/kg CCwere shown in Figure 2.

The calibration curve of CC in rat plasma indicated a high linearity in the range of 1–2500 ng/mL with a correlation coefficient greater than 0.99 (*r*^2^ = 0.9984). There was no significant carry-over under the assay conditions. The LLOQ was 1 ng/mL, which was sufficient for the detection of CC in pharmacokinetic study. The intra-day and inter-day precision (RSD, %) ranged from 4.10% to 5.51% and 5.24% to 6.81%, respectively. The intra-day and inter-day accuracy (RE, %) ranged from −3.28% to 0.56% and −5.83% to −1.44%, respectively (Table 1). The variation of the IS measured value was less than 10%.

The best extraction recovery of CC was obtained using methanol as the protein-precipitating agent. The recoveries of CC at 3, 200 and 2000 ng/mL were 95.22 ± 5.64%, 92.82 ± 8.74% and 92.14 ± 3.45%, respectively. The recovery of the IS was 94.40 ± 4.23%. The results showed that the preparation efficiency of CC and IS in this present study was acceptable.

The matrix effect for CC was evaluated by analyzing three concentrations of QC plasma samples (3, 200, 2000 ng/mL). While the matrix effect for IS was evaluated with a single concentration (100 ng/mL). The average matrix effect values were 94.80 ± 4.06%, 91.49 ± 6.68% and 91.73 ± 3.67% for CC at the low, medium and high QC concentrations, respectively. The matrix effect on IS turned out to be 92.94 ± 5.91% at the tested concentration.

After being placed at 25 °C for 4 h or at −20 °C for two weeks, or undergoing three freeze-thaw (−20 °C to 25 °C) cycles, the results of stability (Table 2) demonstrated that CC was stable.

#### 2.1.3. Pharmacokinetic Study

**Figure 3:**
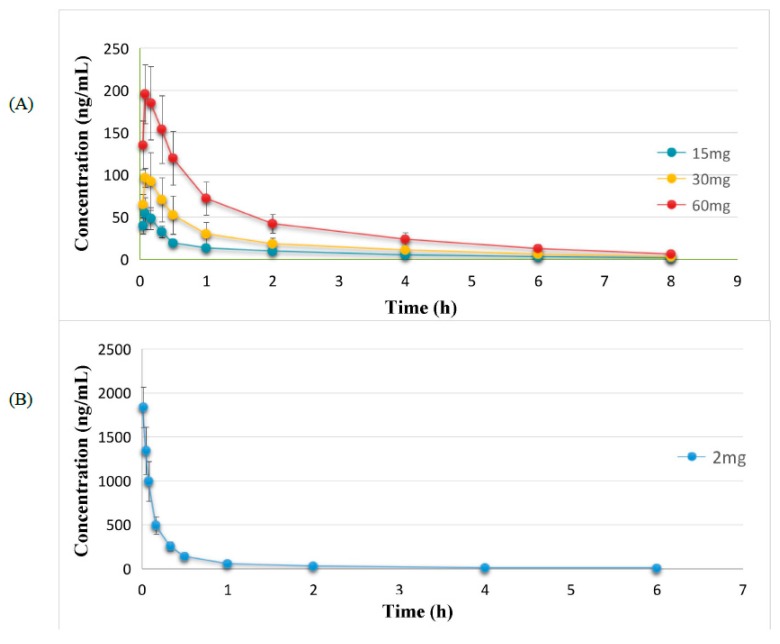
Mean plasma concentration-time profiles of CC in rats after intragastric administration (**A**) and intravenous administration (**B**).

The developed approach was successfully applied to our pharmacokinetic studies after oral administration of 15, 30, 60 mg/kg or intravenous injection of 2.0 mg/kg CC in rats (*n* = 6), respectively. All of the data were calculated with the DAS 3.0. statistical software (Shanghai Bojia Pharmatech Co. Ltd., Shanghai, China). The mean plasma concentration versus time curves and the major pharmacokinetic parameters were shown in Figure 3 and Table 3, respectively. It could be concluded that CC was quickly cleared (clearance, *CL*, 4.87 ± 0.83 L/h/kg; elimination half-life, *t*_1/2_, 1.15 ± 0.20 h) and had high extravascular distribution (apparent volume of distribution, *V_d_*, 8.12 ± 1.97 L/kg) after intravenous administration. As for the three doses of CC after oral administration, the absorption rate of CC was also ultrafast as demonstrated by the fact that CC was detected in plasma at the first blood sampling time (0.05 h) and reached the maximum concentration at 0.083 h. On the other hand, it was concluded that CC was also eliminated rapidly with the *t*_1/2_ from 2.02 h to 2.06 h and *CL* in the range of 201.90–234.19 L/h/kg through the gastrointestinal tract. Whereas the parameter of *V_d_* in the range of 585.34–722.61 L/kg inferred that CC distributed in tissues extensively after gastrointestinal administration. The higher *CL* values and the rapid *T_max_* demonstrated that the oral absorption of CC was poor. Furthermore, the poor absorption as well as the first pass metabolism led to the low AUC values and the poor oral bioavailability (F, 2.01%, 2.13%, and 2.39%, respectively). These observations suggested that this validated analytical LC-MS/MS method was suitable and sufficient for pharmacokinetic study of CC. In short, the characteristic pharmacokinetic properties of CC were rapid oral absorption, high clearance and poor absolute bioavailability. And the short *t*_1/2_ revealed that CC was easily metabolized in vivo. The above characteristics are similar to those of most of the phenolic glycosides reported in the literature [14].

#### 2.1.4. Metabolites Identification of CC

For the determination of fragmentation patterns, the reference compounds of CC were applied for the main MS/MS fragments in UPLC-QTOF-MSE. As Table 4 shows, CC generated a protonated ion [M + Na]^+^ at *m*/*z* 505.1316 and [M + H]^+^ at *m*/*z* 483.1492 and few fragment ions at *m*/*z* 321.1053, 303.0952, 261.0605, 220.9952, 181.0598, 123.1042. The fragment ion at *m*/*z* 321.1053 was produced by the cleavage of the C1″-O bond. By the cleavage of C2-O bond (losing a glucose residue), the fragment ion at *m*/*z* 303.0952 was produced, while the ion at *m*/*z* 261.0605 was attributed to the further loss of a methoxyl and a methyl residue. The fragment ions at *m*/*z* 220.9952 and *m*/*z* 181.0598, both containing the B ring, were produced by the cleavage of the C7-O bond and the cleavage of the C7′-O ester bond, respectively. The last fragment ion at *m*/*z* 123.1042, containing the A ring, was produced by the cleavage of the C1″-O bond and C7-O bond. 

In the UPLC elution, the retention time of CC was 6.80 min, and the molecular ion in the mass spectrum is *m*/*z* 505.1316. Samples of CC-dosed plasma, bile, urine and feces were analyzed in parallel with blank controls through the UPLC-Q/TOF-MS method. Despite the lack of standards for metabolites, their structures could be evaluated on the basis of retention times and mass spectrometry patterns between CC and its product ions.

**Figure 4:**
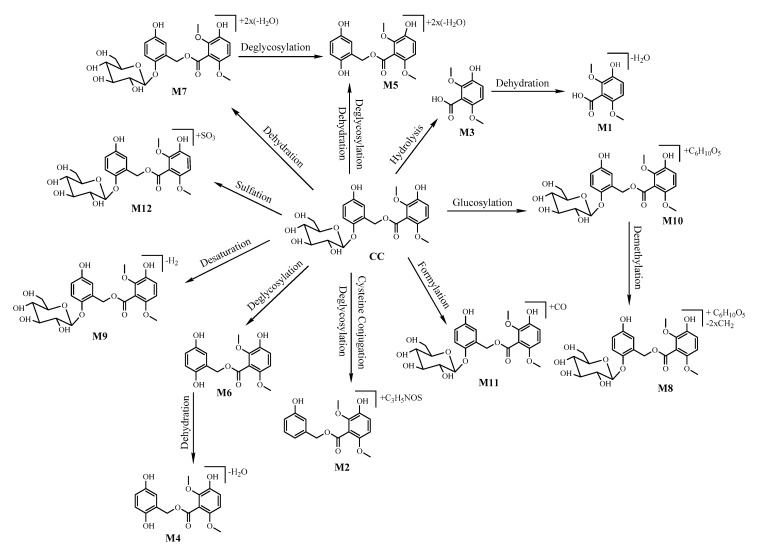
Metabolic profile and proposed metabolic pathways of CC in rats.

Based on certain rules, mass accuracy (±5 ppm), nitrogen rule, isotopic pattern, and double-bond equivalents for instance, the most likely molecular formulas of metabolites were examined. Moreover, according to the MS/MS fragmentation, the tentative chemical structures were determined and common metabolic pathways were profiled. Twelve metabolites including seven phase I metabolites and five phase II metabolites are shown in Figure 4. 

The structures of metabolites were determined and are summarized as follows:

##### Phase I Metabolites

**M3** (C_9_H_10_O_5_), with a retention time of 6.27 min, showed a protonated [M + Na]^+^ ion at *m*/*z* 221.0415, 261 Da lower than that of CC. In addition, the MS fragmentation patterns were analogous to those of the reference substance 3-hydroxy-2,6-dimethoxybenzoic acid, with characteristic fragment ions at *m*/*z* 181.0536, 123.0056, 167.9961, 170.0025. The fragment ion at *m*/*z* 181.0536 was produced by the cleavage of the C7′-OH bond. The ion at *m*/*z* 167.9961 was attributed to the loss of two methyl residues. By the cleavage of C2′-O bond (losing a methoxyl residue) and C1′-C7′, the fragment ion at *m*/*z* 123.0056 was produced. Hence, **M3** was identified as 3-hydroxy-2,6-dimethoxybenzoic acid, the hydrolysis metabolite of CC. 

**M1** was eluted at 4.05 min and presented an accurate protonated ion [M + H]^+^ at *m*/*z* 181.0495 (C_9_H_8_O_4_), 18 Da less than that of **M3**, suggested that **M1** was a dehydration product of **M3**. There were two fragment ions at *m*/*z* 123.0114 and 132.0217 of **M1**. The fragment ion at *m*/*z* 123.0114 was probably produced by loss of a methyl residue and by the cleavage of C1′-C7′, while the fragment ion at *m*/*z* 132.0217 was attributed to the loss of two methyl residues and removal of one molecule of H_2_O from **M1**.

The molecular [M + Na]^+^ ion of **M6** (C_16_H_16_O_7_) was at *m*/*z* 343.0775, 162 Da (Glu) lower than CC, which implies that it was the deglycosylation metabolite of CC. The fragment ions at 123.0328, 181.0326, 220.8950 of **M6** were similar to those of CC. Therefore, **M6** was identified as the aglycone of CC, the hydrolysis metabolite of CC removing a glucose group.

Compound **M4** (C_16_H_14_O_6_), which was of high abundance, gave a [M + H]^+^ molecular ion at *m*/*z* 303.0872 with a retention time of 7.66 min, 18 Da less than **M6**, implying that **M4** could be the result of **M6** losing a molecule of H_2_O. **M4** shared a fragment ion at *m*/*z* 181.0425 similar to CC. Hence, **M4** was identified as the deglycosylation and dehydration product of CC. Another fragment at *m*/*z* 106.0411 containing ring A, was produced by the cleavage of C_2_-O and C_7_-O bond.

**M7** (RT = 14.93 min) gave a [M + H]^+^ molecular ion at *m*/*z* 447.1297 with the formula C_22_H_22_O_10_, which was 36 Da lower than CC. Moreover, the fragment ion at *m*/*z* 267.0531 of **M7** was 36 Da lower than the fragment ion at *m*/*z* 303.0952 of CC. This observation suggests that the metabolite is a dehydration metabolite. Combined with the information of **M5**, it indicated that the dehydration occurred at the parent nucleus. Hence, **M7** was identified as the product of CC losing two molecules of H_2_O. The fragment ion at *m*/*z* 223.0606 of **M7** was produced by the cleavage of the C2-O bond with further loss of one methoxyl group and one methyl group. 

The metabolite denoted by **M5** (C_16_H_12_O_5_) had an accurate mass of *m*/*z* 285.0769 with a [M + H]^+^ ion at a retention time of 9.51 min. These were 162 Da (Glu) lower than **M7**, implying that **M5** is the deglycosylation product of **M7**. In the other hand, it was 18 Da lower than **M4**. The other main fragment ions are at *m*/*z* 257.0453, 105.0336 and 164.9847. Therefore, **M5** was also identified as the a deglycosylation and dehydration metabolite of CC. The fragment ion at *m*/*z* 105.0336 was produced by the cleavage of the C7-O bond of **M5.** The fragment ion at *m*/*z* 164.9847 was produced by the cleavage of the C7′-O ester bond of **M5.** The fragment ion at *m*/*z* 257.0453 was produced by lossing two methyl residues of **M5**.

M9 gave a [M + Na]^+^ molecular ion at *m*/*z* 503.1174 with the formula C_22_H_24_O_12_, detected at a retention time of 18.47 min. **M9** was 2 Da lower than CC, which indicated that desaturation of CC had occurred. The fragment ion at *m*/*z* 450.0755, was produced by loss of one methoxyl of **M9**. While the fragment ion at *m*/*z* 463.1024 was produced from **M9** by loss of one H_2_O.

##### Phase II Metabolites

The protonated metabolite **M12** at *m*/*z* 585.0892 ([M + Na]^+^) eluted at 26.88 min, with a molecular formula C_22_H_26_O_15_S, 80 Da more than that of CC, which implied that sulfation of CC had occurred. The fragment ions at *m*/*z* 303.0779 and *m*/*z* 320.0425 were similar to the fragment ions of CC, which suggested that the sulfation occurred on the glucose group. The actual sulfation position may be further confirmed by the fragment ions at *m*/*z* 259.0245 and *m*/*z* 243.0094. The fragment ions at *m*/*z* 209.0086 might be produced by the cleavage of C7-C1 bond.

**M10** gave a [M + Na]^+^ molecular ion at *m*/*z* 667.1838 with the formula C_28_H_36_O_17_, and was detected at a retention time of 22.41 min. 162 Da (glu) higher than CC, so **M10** should be the glucosylation product of CC. The mass of the fragment at *m*/*z* 465.1102 was 162 Da higher than the characteristic fragment (*m*/*z* 303.0952) of CC, which could be confirmed that glucosylation happened on the parent nucleus.

**M8** eluted at 15.72 min with a [M + Na]^+^ ion at *m*/*z* 639.1541 (C_26_H_32_O_17_), 28 Da lower than that of **M10**, suggesting demethylation had happened in this metabolite. One of the fragments in the MS^2^ spectrum was a [M + Na]^+^ ion at *m*/*z* 437.0844, which was 28 Da less than the fragment at *m*/*z* 465.1102 of **M10**. This observation indicated that the demethylation could occur at the two methyl groups on the ring B of parent nucleus in the combined analysis. The fragment ion at *m*/*z* 179.0473 was the glucose group produced by the cleavage of the C2 bond. 

**M2** was eluted at 4.25 min with a [M + Na]^+^ ion at *m*/*z* 430.0921 (C_19_H_21_NO_7_S). The fragment ions included *m*/*z* 303.0899, 323.0102, 220.0795, indicating that the cysteine conjugation on ring B might occur. The Na adduct ion of the fragment ion at *m*/*z* 323.0102 was produced by C7-O bond cleavage. The other Na adduct ion of the fragment ion at *m*/*z* 220.0795 was produced by the cleavage of the C7-O bond and the cleavage of the ester bond at C3′, while the fragment ion at *m*/*z* 323.0102 was produced by the cleavage of ester bond at C3′ of **M2**. Thus, **M2** was identified as the deglycosylation and cysteine conjugation metabolite. 

The measured [M + H]^+^ *m*/*z* of **M11** was 511.1465, eluted at 22.92 min with a molecular formula C_23_H_26_O_13_, 28 Da higher than CC, which indicated that it was a formylated product of CC. Abundant daughter ions at *m*/*z* 331.0715 in the MS^2^ spectrum, which were 28 Da higher than the fragment ion at *m*/*z* 303.0952 of CC, support the notion that decarboxylation had occured to the parent nucleus. The fragment ion at *m*/*z* 331.0715 was conjectured to be the formylated product of the typical fragment of CC at *m*/*z* 303.0952.

In conclusion, an effective strategy using UPLC-Q-TOF-MSE coupled with the UNIFI 1.7.0 software (Waters, Manchester, UK) for quick characterising and identificating metabolites of CC was developed. In our study, the pathways of Phase I metabolites included dehydration, hydrolysis, deglycosylation, desaturation, and for Phase II metabolites they were sulfation, glucosylation, cysteine conjugation, formylation, demethylation. These results could provide a theoretical foundation for understanding the pharmacological effects and metabolic processes of CC.

## 3. Discussion

Targeting at exploring the characteristics of the phenolic glycosides, the pharmacokinetics and metabolism of CC were investigated. First of all, a UPLC-MS/MS method with high sensitivity (1 ng/mL) and a short running time (3.5 min), was validated and used for the pharmacokinetic study. Secondly, the pharmacokinetic analysis elucidated that CC was rapidly absorbed and quickly eliminated, which is similar to curculigoside A, but CC showed better bioavailability (2.39%) than that of curculigoside A (0.38%) [15], which might be the reason for the better activity of CC. Finally, the major metabolites of Phase I and Phase II reactions and the proposed metabolic pathways of CC were both profiled. Furthermore, the results of metabolite identification demonstrated that CC was mainly metabolized in the liver, since all metabolites could be found in bile, urine and feces samples. Considering the results acquired in this research, we deduce that the poor oral bioavailability of CC in rats might be related to hepatic microsomes. Since CC acted as an antioxidant, it is worth mentioning the quinone metabolites, which were not identified under the screening conditions used in the samples of healthy rats. The reason might be as follows: in order to obtain the main metabolites with high response and good mass accuracy, the metabolites with worse mass accuracy (out of the range of ±5 ppm) and low response (below 5000) were filtered firstly before the identification of metabolites. During this step, quinones might be filtered due to their low content. In future studies, the model rats with peroxidation injury may be used to investigate possible quinone metabolites. Besides the common pathways of curculigoside A, such as dehydration and deglycosylation, four other metabolic pathways including formylation, desaturation, cysteine conjugation and sulfonation also had been discovered in CC. More importantly, the -OH at C-3′ still existed in all the metabolites, which further confirmed that the -OH at C-3′ might be the functional group of CC, but the low absolute bioavailability might limit its further application. Future studies should focus on structure modification to improve the bioavailability.

## 4. Experimental

### 4.1. Materials and Reagents

**Figure 5:**
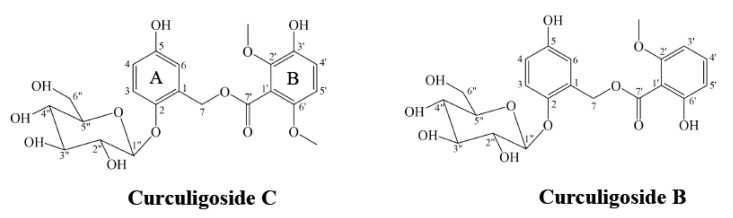
Chemical structures of CC and curculigoside B.

CC (purity: 99.1%) was isolated from Curculiginis rhizoma in our laboratory and identified by HR-MS and Nuclear Magnetic Resonance (NMR) spectroscopy. Its purity was determined by using high performance liquid chromatography (HPLC). Internal standard (IS, Curculigoside B) was provided by Wuhan Tian Zhi Biotechnology Co., Ltd. (Hubei, China). 3-Hydroxy-2,6-dimethoxybenzoic acid was obtained from HE Chemical Co., Ltd. (Jiangsu, China). The chemical structures of CC and Curculigoside B are shown in Figure 5. UPLC-MS pure grade methanol and acetonitrile were purchased from Fisher (Geel, Belgium). Formic acid was acquired from the Sigma-Aldrich Company (St. Louis, MI, USA). Deionized water was purified with a Millipore water purification system (Millipore, Billerica, MA, USA). All other chemicals were of analytical grade. Blank rat plasma samples (drug-free and anti-coagulated with heparin sodium) were prepared by our group.

### 4.2. Animals and Drug Administration

All animal experiments were approved (permit number: 20180057) by the Review Committee of Animal Care and Use of Jilin University according to ethical principles for animal use and care. Wistar rats (200 ± 20 g) were purchased from Changchun Yisi Laboratory Animal Co. Ltd. (Changchun, China), and were housed under standard conditions in a controlled breeding room (12 h light/dark cycle, temperature: 22 ± 2 °C, relative humidity: 55 ± 5%). All rats were fed with standard laboratory food and water *ad libitum*, except during a fast period prior to the experiments. For the pharmacokinetic study, the rats were divided into four groups (*n* = 6, 3 males and 3 females): (1) CC (15 mg/kg, i.g.), (2) CC (30 mg/kg, i.g.), (3) CC (60 mg/kg, i.g.) and (4) CC (2.0 mg/kg, i.v.). While for the metabolism study, CC (60 mg/kg, i.g.) was gastrointestinally administrated to achieve higher concentrations of the metabolites. 

### 4.3. Sample Preparation

#### 4.3.1. Pharmacokinetic Study

The stock solution of CC and IS was prepared by dissolving the accurately weighed reference compound in methanol at 1.00 mg/mL, respectively. The blank rat plasma was prepared as follows: blood samples were collected from the abdominal aorta immediately and incubated at 37 °C for half an hour, then a 15 min centrifugation at 4000 rpm was carried out to separate the plasma, the supernatant was kept at −20 °C until analysis. After dilutions were spiked with blank rat plasma, the calibration curve for CC was plotted to produce the points equivalent to 1, 2.5, 5, 25, 50, 250, 500, 2500 ng/mL. With the same treatment as calibration standards, 3, 200 and 2000 ng/mL of quality control (QC) samples were prepared independently. All samples were prepared freshly before analysis.

The whole blood (150 μL) from the orbital venous plexus was collected into heparinized microcentrifuge tubes at 0.05, 0.083, 0.167, 0.333, 0.5, 1, 2, 4, 6, 8 h after gastrointestinal administration, and 0.017, 0.05, 0.083, 0.167, 0.333, 0.5, 1, 2, 4, 6 h after intravenous administration. After a 15 min centrifugation step at 4000 rpm and 4 °C of the blood collected, plasma was obtained. 

All samples were kept at −20 °C before analysis. Frozen plasma samples were thawed to room temperature and vortexed. CC was extracted from plasma only by single step precipitation method. Both 50 μL of each rat plasma sample and 500 μL of methanol containing 100 ng/mL IS were transferred into 2.0 mL microcentrifuge tubes, and then extracted by vortexing for 3 min to deproteinize the endogenous protein. After a 10 min centrifugation step at 10,000 rpm and 4 °C, the supernatant was collected and transferred into vials, and 2 μL of it was injected into UPLC-MS system for quantification. All prepared samples were stored in the autosampler at 10 °C until injection. 

#### 4.3.2. Metabolism Study

After intragastric administration, blood samples were collected at 0.25, 0.5, 0.75, 1, 1.5, 2, 2.5, 3, 6, 12 h. After vortexing 500 μL of pooled plasma sample with 2 mL of methanol, the mixtures were centrifuged at 10,000 rpm and 4 °C for 10 min to obtain the clear supernatant, which was evaporated with N_2_. Then, the residue was reconstituted in 100 μL of methanol.

To obtain bile samples, a plastic cannula was surgically inserted into the bile ducts to collect the bile through an abdominal incision made after rats were anesthetized with intraperitoneal injection of urethane (1.0 g/kg). Blank bile was collected for 2 h before dosed, and bile samples were collected for 12 h after administration. While the blank and dosed samples of urine and feces were obtained respectively with metabolic cages equipped with separator prior to drug administration or 18 h after oral administration. The mixture was centrifuged for 10 min at 10,000 rpm and 4 °C after diluting pooled urine and bile sample with triple volume of methanol. The obtained supernatant was evaporated by N_2_ at room temperature after transferred into another tube. The dried residue of urine or bile was dissolved in 50 μL of methonal, respectively. Finally, the supernatant being centrifuged for 10 min at 10,000 rpm and 4 min was applied to UPLC-MS/MS analysis.

Faeces samples were dried and crushed into powder before being stored. Each 10 mg of faeces powder was added with 1 mL of methanol and was extracted for 30 min using an ultrasonic ice-water bath. After centrifugation at 10,000 rpm and 4 °C for 10 min, the supernatant was transferred to a clean tube and then evaporated to dryness under a stream of N_2_. The residue reconstituted with 500 μL methanol was centrifuged at 10,000 rpm and 4 °C for 10 min. All biological samples were stored at −20 °C.

### 4.4. Instruments and Experimental Conditions

#### 4.4.1. LC-MS/MS Conditions

Quantification of CC was performed on an Acquity UPLC unit coupled with a XEVO TQ-S mass spectrometer equipped with an electrospray ionization (ESI) source (Waters Co.). Separation was achieved using a Waters BEH C18 UPLC column (2.1 mm × 50 mm, 1.7 μm) at 40 °C. The optimized method used binary gradient mobile phases with 0.1% formic acid in acetonitrile as mobile phase A and 0.1% formic aqueous solution as mobile phase B (0–2 min, 20% → 30% A; 2–2.5 min, 30% → 20% A; 2.5–3.5 min, 20% A). A flow rate of 0.3 mL/min was used with 2 μL of injection volume. Chromatography of the CC and IS was performed within 3.5 min. CC was quantified using multiple reaction monitoring (MRM) in the positive ESI mode and the optimized MS parameters were set as follows: capillary voltage floating at 3200 V; 150 °C for source temperature; 300 °C for desolvation temperature; 150 L/h for flow of cone gas; 800 L/h for flow of desolvation gas; 0.15 mL/min for flow of collision gas; nebulizer gas flow at 7 bar; cone voltage at 70 and 48 V for CC and IS, respectively; Collision energy at 20 and 18 eV for CC and IS, respectively. Data acquisition and processing were operated through Masslynx V4.1 workstation.

#### 4.4.2. UPLC-QTOF/MS Conditions

Metabolic analysis was conducted on a Waters ACQUITY UPLC System coupled with a Xevo G2-S Q-TOF mass spectrometer in ESI^+^ mode. A Waters UPLC BEH C_18_ column (2.1 mm × 50 mm, 1.7 μm) was used with the following parameter settings: 30 °C for the column temperature; 0.4 mL/min for flow rate; 10 μL for injection volume; 4 °C for the autosampler temperature; and the mobile phase consisted of 0.1% formic aqueous solution (A) and 0.1% formic acid in acetonitrile (B) in proportions adjusted through a gradient elution programme as follows: 0–2 min, 10% B; 2–26 min, 10% → 100% B; 26–28 min, 100% B; 28–28.1 min, 100% → 10% B, 28.1–30 min, 10% B. The following optimized conditions were carried out: 2.6 kV for capillary voltage; 40 V for cone voltage; 120 °C for source temperature; 300 °C for desolvation temperature; 50 L/h for the flow of cone gas; 800 L/h for the flow of desolvation gas. MSE acquisition mode is an intelligent approach, which collects exact-mass precursor and product ion information from a single injection in a data-independent manner. It could parallel alternate scans at either low collision energy to obtain precursor ion information, or high collision energy to obtain full-scan accurate mass fragment, precursor ion and neutral loss information. In doing so, it excludes false positive results; requires no “knowledge” of the ions to be fragmented; runs parallel precursor, product ion, and neutral loss analyses; and streamlines data interrogation and reporting. In MS^E^ mode, the collision energy of low energy function and high energy function was set at 6 V and 20–40 V, respectively. The mass spectrometer was calibrated over a range of 100–1000 Da with sodium formate to ensure mass accuracy. Leucine-enkephalin (*m*/*z* 556.2771) was used as the lockmass at a concentration of 200 ng/mL and flow rate of 10 μL/min. Masslynx V4.1 workstation in continuum mode was used for data collection. Metabolic characterization of CC was analyzed using UNIFI 1.7.0 software (Waters, Manchester, UK).

### 4.5. Method Validation

According to the Bioanalytical Method Validation Guideline (Chinese Pharmacopoeia 2015, Vol. 4) and Drug Non-Clinical Pharmacokinetic Study Technical Guideline (China Food And Drug Administration 2014), specificity, accuracy and precision, linearity, LLOQ, extraction recovery and matrix effect had been validated in this present study.

Specificity was investigated by analyzing chromatograms of blank rat plasma, plasma spiked with CC and IS, and experimental plasma sample following dosing of CC. Linearity was analyzed through weighted regression (1/*x*^2^) of peak area ratios (*y*) of CC to IS versus nominal concentration (*x*) in plasma. The blank plasma has been run after the determination of upper concentration (2500 ng/mL) of the standard curve aiming at determine the carry-over in the assay condition. Precision and accuracy were evaluated by analyzing three concentration levels (3, 200 and 2000 ng/mL) of QC samples. The intra-, inter-day precision and accuracy were assessed by determining at three QC levels (3, 200 and 2000 ng/mL) in replicates of six QC samples. The precision was expressed as relative standard deviation (RSD, %) and the accuracy was expressed as relative error (RE, %). Both of the values for QC concentrations within 15% were acceptable. The lowest limit of quantification (LLOQ) was the lowest concentration on calibration curve, with acceptable precision (RSD ≤ 20%) and accuracy (RE ≤ ±20%). The extraction recovery of CC from rat plasma at three QC concentrations was assessed by comparing peak areas of the QC samples pre-spiked in blank plasma (A) with those of post-extracted blank plasma spiked at the same concentration (B). The matrix effect of CC from rat plasma was evaluated by comparing the peak areas of post-extracted spiked rat plasma (B) with those of equivalent concentrations of the pure authentic standard at three QC concentrations (C). The recovery and matrix effect of IS were evaluated at 100 ng/mL in the same way as CC. Stability in plasma samples was investigated by analyzing QC samples (*n* = 6) under various storage conditions: (1) at room temperature for 4 h; (2) at −20 °C for two weeksl; (3) subjected to three complete freeze/thaw cycles (from −20 °C to room temperature) on consecutive days; (4) stored in the stock solutions (4 °C) and in methanol with plastic autosampler vials for 16 h at 10 °C. 

### 4.6. Pharmacokinetic Study

The concentration versus the time curve was plotted. The pharmacokinetic parameters, including the maximum plasma concentration (*C_max_*) and the time to reach the peak concentration (*T_max_*) were obtained in rat plasma after intragastric administration, elimination half-life (*t*_1/2_), area under the plasma concentration–time curve (*AUC*), were analyzed through a non-compartmental pharmacokinetic analysis carried out by Drug and Statistics (DAS) 3.0 pharmacokinetic software programme (Mathematical Pharmacology Professional Committee of China, Shanghai, China). The results were presented as the mean ± standard deviation. Absolute bioavailability (F%) was determined through the equation (AUC_p.o._ × Dose_i.v._)/(AUC_i.v._ × Dose_p.o._) × 100%.

## 5. Conclusions

In the present assay, the pharmacokinetics and metabolism of curculigoside C (CC) were investigated for the first time. In the pharmacokinetic analysis, a rapid, sensitive and reproducible UPLC-ESI-MS/MS quantification method was developed and validated for determination of CC in rat plasma samples. The sample preparation process was simple and the analysis time was just 3.5 min. The method was then successfully applied to the pharmacokinetic study after intravenous administration of 2.0 mg/kg CC and oral administration of 15, 30 and 60 mg/kg CC to rats, respectively. The results showed that CC exhibited rapid oral absorption (*T_max_* = 0.11 h), high elimination (*t*_1/2_ = 2.01 h) and poor absolute bioavailability (2.39%). A metabolic investigation of CC was conducted through UPLC-QTOF-MS^E^. The major metabolites and metabolic pathways were all characterized. In general, CC, another phenolic glycoside in comparation with curculigoside A, has the similar pharmacokinetic parameters and metabolic pathways, but better absolute bioavailability. These findings provide foundation for pharmacological research and development of CC.

## Figures and Tables

**Table 1:** Intra- and inter-day precision and accuracy of CC in rat plasma.

Concentration (ng/mL)	Intra-Day Measured Concentration (ng/mL)	Precision (RSD, %)	Accuracy (RE, %)	Inter-Day Measured Concentration (ng/mL)	Precision (RSD, %)	Accuracy (RE, %)
3	3.02 ± 0.12	4.10	0.56	2.96 ± 0.15	5.24	−1.44
200	195.5 ± 9.40	4.81	−2.25	188.33 ± 12.82	6.81	−5.83
2000	1934.33 ± 106.49	5.51	−3.28	1890 ± 123.94	6.56	−5.50

**Table 2:** The stability of curculigoside C and IS in plasma (*n* = 6).

	Concentration		Mean ± SD	Accuracy
	(ng/mL)		(ng/mL)	(%)
Short-term stability	CC	3	2.91 ± 0.13	−3.00
(25 °C, 4 h)		200	185.5 ± 8.60	−7.25
		2000	1882.50 ± 117.78	−5.88
	IS	100	92.83 ± 3.31	−7.17
Long-term stability	CC	3	2.84 ± 0.09	−5.22
(10 °C, 16 h)		200	184.33 ± 12.23	−7.83
		2000	1870.50 ± 110.70	−6.48
	IS	100	92.50 ± 5.68	−7.50
Freezing stability	CC	3	2.945 ± 0.11	−1.83
(−20 °C, 14 d)		200	193.83 ± 10.23	−3.08
		2000	1908.17 ± 103.51	−4.59
	IS	100	94.00 ± 5.33	−6.00
	CC	3	2.87 ± 0.11	−4.33
(3 freeze-thaw cycles)		200	185.17 ± 9.50	−7.42
		2000	1889.67 ± 124.23	−5.52
	IS	100	90.83 ± 5.19	−9.17

**Table 3:** Pharmacokinetic parameters of CC in rat plasma after intragastric administration.

Dose (mg/kg)	*t*_1/2_ (h)	*T_max_* (h)	AUC_(0–60)_ (μg/L/h)	AUC_(0–∞)_ (μg/L/h)	F (%)	Vd (L/kg)	CLz (L/h/kg)
15	2.022 ± 0.184	0.106 ± 0.149	62.731 ± 10.149	66.310 ± 10.563	2.01	673.157 ± 116.509	231.036 ± 36.69
30	2.061 ± 0.325	0.111 ± 0.043	133.17 ± 48.434	140.656 ± 48.335	2.13	722.605 ± 316.306	234.188 ± 74.864
60	2.048 ± 0.227	0.111 ± 0.043	299.155 ± 80.954	316.980 ± 91.704	2.39	585.344 ± 124.289	201.905 ± 55.193
2.0 (i.v)	1.153 ± 0.203	-	416.700 ± 70.401	420.700 ± 70.690	-	8.122 ± 1.973	4.869 ± 0.834

**Table 4:** Metabolites of CC detected and structurally characterized on a UPLC-Q-TOF-MSE.

No.	RT (min)	Formula	Measured Mass (*m*/*z*)	Calculate Mass (*m*/*z*)	Error (ppm)	Fragment Ions	Molecular Ion	Source
CC	6.71	C_22_H_26_O_12_	505.1316	482.1424	−1.5	321.1053, 303.0952, 261.0605, 220.9952, 181.0598, 123.1042	[M + Na]^+^	B,P,U
M1	4.05	C_9_H_8_O_4_	181.0495	180.0423	−1.7	132.0217, 123.0114	[M + H]^+^	B,P,U
M2	4.25	C_19_H_21_NO_7_S	430.0921	407.1039	−3.4	303.0899, 323.0102, 220.0795	[M + Na]^+^	B,U
M3	6.27	C_9_H_10_O_5_	221.0415	198.0528	−3.0	123.0056, 167.9961, 170.0025, 181.0536	[M + Na]^+^	B,F
M4	7.66	C_16_H_14_O_6_	303.0872	302.0790	2.3	181.0425, 132.9930, 106.0411	[M + H]^+^	B,P
M5	9.51	C_16_H_12_O_5_	285.0769	284.0685	3.2	257.0453, 105.0336, 164.9847	[M + H]^+^	B,P
M6	10.29	C_16_H_16_O_7_	343.0775	320.0896	−4.4	123.0328, 181.0326, 220.8950	[M + Na]^+^	B,U,F
M7	14.93	C_22_H_22_O_10_	447.1297	446.1213	2.0	267.0531, 164.9847, 223.0606	[M + H]^+^	B,P,U
M8	15.72	C_26_H_32_O_17_	639.1541	616.1639	1.5	437.0844, 179.0473, 123.0320	[M + Na]^+^	B,P,U,F
M9	18.47	C_22_H_24_O_12_	503.1174	480.1268	2.7	463.1024, 450.0755, 220.0650	[M + Na]^+^	B,U,F
M10	22.41	C_28_H_36_O_17_	667.1838	644.1952	−1.1	612.1283, 465.1102, 303.0946	[M + Na]^+^	B,P,U,F
M11	22.92	C_23_H_26_O_13_	511.1465	510.1373	3.1	123.0306, 181.0726, 331.0715	[M + H]^+^	B,F
M12	26.88	C_22_H_26_O_15_S	585.0892	562.0992	1.2	209.0086, 190.9980, 303.0779, 320.0425, 259.0245, 243.0094	[M + Na]^+^	B,F

P: plasma samples, B: bile samples, U: urine samples, F: feces samples.
